# A clustering-based sampling method for miRNA-disease association prediction

**DOI:** 10.3389/fgene.2022.995535

**Published:** 2022-09-13

**Authors:** Zheng Wei, Dengju Yao, Xiaojuan Zhan, Shuli Zhang

**Affiliations:** ^1^ School of Computer Science and Technology, Harbin University of Science and Technology, Harbin, China; ^2^ College of Computer Science and Technology, Heilongjiang Institute of Technology, Harbin, China

**Keywords:** miRNA-disease association, ensemble learning, clustering, sampling, computational methods

## Abstract

More and more studies have proved that microRNAs (miRNAs) play a critical role in gene expression regulation, and the irregular expression of miRNAs tends to be associated with a variety of complex human diseases. Because of the high cost and low efficiency of identifying disease-associated miRNAs through biological experiments, scholars have focused on predicting potential disease-associated miRNAs by computational methods. Considering that the existing methods are flawed in constructing negative sample set, we proposed a clustering-based sampling method for miRNA-disease association prediction (CSMDA). Firstly, we integrated multiple similarity information of miRNA and disease to represent miRNA-disease pairs. Secondly, we performed a clustering-based sampling method to avoid introducing potential positive samples when constructing negative sample set. Thirdly, we employed a random forest-based feature selection method to reduce noise and redundant information in the high-dimensional feature space. Finally, we implemented an ensemble learning framework for predicting miRNA-disease associations by soft voting. The Precision, Recall, F1-score, AUROC and AUPR of the CSMDA achieved 0.9676, 0.9545, 0.9610, 0.9928, and 0.9940, respectively, under five-fold cross-validation. Besides, case study on three cancers showed that the top 20 potentially associated miRNAs predicted by the CSMDA were confirmed by the dbDEMC database or literatures. The above results demonstrate that the CSMDA can predict potential disease-associated miRNAs more accurately.

## 1 Introduction

MicroRNAs (miRNAs) are a kind of non-coding RNAs with a length of 20–24 nucleotides, which play a critical role in gene expression regulation (Lee et al., 1993; Wightman et al., 1993; He & Hannon, 2004). Accumulating evidences have showed that the dysregulation of miRNA is associated with human complex diseases (Hwang & Mendell, 2006; Mattick & Makunin, 2006; Jonas & Izaurralde, 2015). Wang et al. have proved that the expression level of hsa-mir20b-5p is associated with the pathogenesis of Alzheimer’s disease (Wang et al., 2022). Taverner et al. have proposed that microRNA-425–5p and microRNA-451 can be used as the risk biomarkers of cardiovascular disease (Taverner et al., 2021). Ma et al. have showed that the overexpression of microRNA-10b promotes invasion and metastasis of mammary tumor cells (Ma et al., 2007). Hashimoto et al. have demonstrated that the abnormal expression of miR-1307–3p in human serum is associated with a variety of malignant tumors (Hashimoto et al., 2021). Therefore, accurately identifying disease-associated miRNAs can facilitate the study of the mechanism of miRNA in complex diseases. To guide complex biological experiments, many computational models have been developed for predicting miRNA-disease associations (Chen et al., 2019a).

Thus far, scholars have proposed a series of network-based miRNA-disease association prediction models (Bandyopadhyay et al., 2010). Jiang et al. integrated a human miRNA-phenome network and a miRNA function-related network for predicting disease-associated miRNAs (Jiang et al., 2010). Shi et al. mapped the pathogenic disease genes and miRNA target genes into the protein-protein interaction network, and employed the random walk with restart to identify miRNA-disease associations (Shi et al., 2013). Zeng et al. implemented a structural perturbation approach for miRNA-disease association prediction on a bilayer network which integrated the known miRNA-disease associations and miRNA (disease) similarity network (Zeng et al., 2018). Xiao et al. first calculated the weighted K nearest neighbor profiles of miRNAs and diseases, and then used graph regularized matrix factorization to predict miRNA-disease associations (Xiao et al., 2018). Zhong et al. proposed a global method based on non-negative matrix factorization, which could simultaneously predict all disease-related miRNAs (Zhong et al., 2018). Ma et al. presented a miRNA-disease association prediction model which did not depend on any known miRNA-disease associations (Ma et al., 2019). Li et al. constructed a heterogeneous bilayer network by integrating similarity networks and interaction network, and then utilized the algorithm faster randomized partial matrix completion to infer latent disease-lncRNA associations (Li et al., 2019). Yu et al. proposed a knowledge-driven method to predict disease-miRNA associations (KDFGMDA) (Yu et al., 2022). Based on dynamic neighborhood regularized logistic matrix factorization, Yan et al. proposed a method (DNRLMF-MDA) to predict miRNA-disease associations (Yan et al., 2019). Qu et al. proposed a biased random walk computational method for miRNA-disease association prediction (BRWRMHMDA), which was restarted on multilayer heterogeneous networks (Qu et al., 2021). Jiang and Zhu proposed a model of decision template-based miRNA-disease association prediction (DTMDA) (Jiang & Zhu, 2020).

In recent decades, dozens of miRNA-disease association prediction models based on machine learning have been proposed. One of the major challenges facing these models is how to construct negative samples set. Yao et al. implemented an improved random forest-based model for miRNA-disease association prediction (IRFMDA) which constructed negative samples by randomly combining miRNAs and diseases (Yao et al., 2019). Zhao et al. proposed an adaptive boosting model (ABMDA) which employed the k-means algorithm to cluster the unlabeled samples and selected negative samples randomly from each cluster (Zhao et al., 2019). Zhou et al. designed a miRNA-disease association prediction model based on gradient boosting decision tree and logistic regression (GBDT-LR) which applied the k-means algorithm to cluster the unlabeled samples and extracted negative samples from each cluster by the ratio of the size of each cluster to the entire unlabeled sample set size (Zhou et al., 2020). Li et al. proposed a graph auto-encoder-based miRNA-disease association prediction model (GAEMDA) which randomly selected 5,430 unlabeled samples as negative samples (Li et al., 2021). Chen et al. proposed an anti-noise miRNA-disease association prediction algorithm (ANMDA) which applied the k-means algorithm to cluster the unlabeled samples and selected negative samples equally from each cluster to reduce the noise (Chen et al., 2021). Dai et al. presented a resampling-based ensemble framework (ERMDA) which constructed multiple balanced training subsets by resampling and obtained the final prediction result by soft voting strategy (Dai et al., 2022). Liu et al. proposed a new novel method via deep forest ensemble learning based on autoencoder (DFELMDA) to predict miRNA-disease associations (Liu et al., 2022). Chen et al. presented a model of extreme gradient boosting machine for miRNA-disease association (EGBMMDA), which calculated the statistical measures and matrix factorization results for each miRNA-disease pair to form an information feature vector (Chen et al., 2018). The above methods inevitably introduced potential positive samples into negative sample set, which limited the prediction performance of these models (Rayhan et al., 2017).

In this paper, we proposed a novel clustering-based sampling method for miRNA-disease association prediction (CSMDA) which could construct more reliable negative sample set. Firstly, the CSMDA integrated a variety of similarity information of miRNA and disease to represent the feature vector of miRNA-disease pairs. Secondly, the CSMDA constructed negative sample set based on MiniBatchKMeans clustering to reduce the proportion of potentially positive samples in the negative samples set. Thirdly, the CSMDA generated numerous training subsets through multiple rounds of sampling on the negative sample set to reduce the bias caused by single small-scale sampling. Fourthly, the CSMDA applied a random forest-based feature selection approach to reduce noise and redundant information in the high-dimensional feature space. Finally, a set of base classifiers were trained on the training subsets after feature selection and the final prediction result was obtained by soft voting. The Precision, Recall, F1-score, AUROC and AUPR of the CSMDA achieved 0.9676, 0.9545, 0.9610, 0.9928 and 0.9940 under 5-fold cross-validation, which was significantly higher than that of the existing methods. Besides, case study on three cancers showed that all the top 20 miRNAs predicted to be most likely associated with these cancers by the CSMDA were confirmed by the dbDEMC database or literatures.

## 2 Materials and methods

### 2.1 Experimentally confirmed miRNA-disease associations

Experimentally confirmed 5,430 miRNA-disease associations were obtained from the HMDD (Human microRNA Disease Database) (Li et al., 2014), including 495 miRNAs and 383 diseases. Here, we stored these miRNA-disease associations by a matrix $MD_{N_{m}\times N_{d}}$ , which was defined as:

MD(m(i),d(j))={1,miRNA m(i)and disease d(j)are verified to be related0,miRNA m(i)and disease d(j)are not verified to be related (1)

Here, $N_{m}$ and $N_{d}$ represent the number of miRNAs and diseases, respectively.

### 2.2 Disease semantic similarity

The descriptors of 383 diseases mentioned above were obtained from the MeSH (Medical Subject Headings) database and Directed Acyclic Graphs (DAGs) for each disease were constructed by the previous methods (Wang et al., 2010; Xuan et al., 2013). In a DAG (D) , the nodes represent disease D and its ancestral nodes, and the directed edges represent the relationship of diseases. The semantic contribution of disease d to disease D in DAG (D) was defined as follows:

$$D1_{D}\left(d\right)=\left\{ \begin{array}{cc} 1, & d=D\\ \max\left\{\Delta \times D1_{D}\left(d^{\prime }\right)d^{\prime }\in children\;of\;d\right\}, & d\neq D \end{array}\right.$$ (2)

Here, Δ is the semantic contribution factor. As the distance between D and other diseases in DAG(D) increases, the semantic contribution of these diseases will decrease. Then, the semantic value of disease D was defined as follows:

$$\begin{array}{c} DV1\left(D\right)=\underset{d\in T\left(D\right)}{\sum }D1_{D}\left(d\right) \end{array}$$ (3)

Here, T(D) represents the disease D and its all ancestral nodes. For two diseases, d(k) and d(l) , the disease semantic similarity between them was defined as follows:

$$\begin{array}{c} SS1\left(d\left(i\right),d\left(j\right)\right)=\frac{\sum_{t\in T\left(d\left(i\right)\right)\cap T\left(d\left(j\right)\right)}\left(D1_{d\left(i\right)}\left(t\right)+D1_{d\left(j\right)}\left(t\right)\right)}{DV1\left(d\left(i\right)\right)+DV1\left(d\left(j\right)\right)} \end{array}$$ (4)

Considering two different diseases in the same layer of a DAG (D) , if the occurrence rate of one disease is different from another, their semantic contribution to disease D should be different. Inspired by Xuan et al. (Xuan et al., 2013), another way to calculate the semantic contribution of disease d in DAG (D) to disease D was defined as follows:

$$\begin{array}{c} D2_{D}\left(d\right)=-\log\frac{the\;number\;of\;DAGs\;including\;d}{the\;number\;of\;disease} \end{array}$$ (5)

Similarly, the disease semantic value DV2(D) of disease D was defined as follows:

$$\begin{array}{c} DV2\left(D\right)=\underset{d\in T\left(D\right)}{\sum }D2_{D}\left(d\right) \end{array}$$ (6)

Then, the disease semantic similarity between disease d(i) and disease d(j) was defined as follows:

$$\begin{array}{c} SS2\left(d\left(i\right),d\left(j\right)\right)=\frac{\sum_{t\in T\left(d\left(i\right)\right)\cap T\left(d\left(j\right)\right)}\left(D2_{d\left(i\right)}\left(t\right)+D2_{d\left(j\right)}\left(t\right)\right)}{DV2\left(d\left(i\right)\right)+DV2\left(d\left(j\right)\right)} \end{array}$$ (7)

Finally, we combined the above two methods to calculate the disease semantic similarity of disease d(i) and d(j) as follows:

$$\begin{array}{c} SS\left(d\left(i\right),d\left(j\right)\right)=\frac{SS1\left(d\left(i\right),d\left(j\right)\right)+SS2\left(d\left(i\right),d\left(j\right)\right)}{2} \end{array}$$ (8)

### 2.3 Gaussian interaction profile kernel similarity for diseases

Based on the assumption that miRNAs with similar functions tend to be related to diseases with similar phenotypes (van Laarhoven et al., 2011), Gaussian interaction profile kernel (GIPK) similarity for diseases was introduced to represent the relationship between diseases from another perspective. Here, let IP (d(i)) represent the i th column vector of the miRNA-disease association matrix MD , which denotes whether there are verified associations between disease d(i) and each miRNA. Then, the GIPK similarity of disease d(i) and d(j) was defined as follows:

$$\begin{array}{c} GD\left(d\left(i\right),d\left(j\right)\right)=\exp\left(-\gamma_{d}IP\;\left(d\left(i\right)\right)-IP\;{\left(d\left(j\right)\right)}^{2}\right) \end{array}$$ (9)

In Eq. 9, parameter $\gamma_{d}$ controls the kernel bandwidth and was calculated by the following formula:

$$\begin{array}{c} \gamma_{d}=\frac{\gamma_{d}^{\prime }}{\frac{1}{N_{d}}\sum_{i=1}^{N_{d}}IP\;{\left(d\left(i\right)\right)}^{2}} \end{array}$$ (10)

According to the previous study (Chen & Yan, 2013; Chen et al., 2016), $\gamma_{d}^{\prime }$ was set to 1 here.

### 2.4 Integrated similarity of diseases

Since there may be no semantic similarity between two diseases, we integrated semantic similarity and GIPK similarity of disease here. Inspired by previous works (Dai et al., 2022), the integrated disease similarity between d(i) and d(j) was defined as follows:

$$\begin{array}{c} IDS\left(d\left(i\right),d\left(j\right)\right)=\left\{ \begin{array}{c} SS\left(d\left(i\right),d\left(j\right)\right),\;SS\left(d\left(i\right),d\left(j\right)\right)\neq 0\\ GD\left(d\left(i\right),d\left(j\right)\right),\;SS\left(d\left(i\right),d\left(j\right)\right)=0 \end{array}\right. \end{array}$$ (11)

### 2.5 MiRNA functional similarity

Based on the hypothesis that miRNAs with similar functions tend to be associated with diseases with similar phenotypes, miRNA functional similarity can be calculated (Wang et al., 2010). Here, we directly obtained miRNA functional similarity from the MISIM database (http://www.cuilab.cn/fi les/images/cuilab/misim.zip) and represented them by FS(m(i),m(j)) .

### 2.6 Gaussian interaction profile kernel similarity for miRNAs

Similar to disease, the GIPK similarity between miRNA m(i) and m(j) was defined as follows:

$$\begin{array}{c} GM\left(m\left(i\right),m\left(j\right)\right)=\exp\left(-\gamma_{m}IP\;\left(m\left(i\right)\right)-IP\;{\left(m\left(j\right)\right)}^{2}\right) \end{array}$$ (12)

$$\begin{array}{c} \gamma_{m}=\frac{\gamma_{m}^{\prime }}{\frac{1}{N_{m}}\sum_{i=1}^{N_{m}}IP\;{\left(m\left(i\right)\right)}^{2}} \end{array}$$ (13)

Here, IP (m(i)) represent the i th row vector of miRNA-disease associations matrix MD , which indicates whether there are verified associations between miRNA m(i) and each disease. Inspired by previous works (Chen & Yan, 2013; Chen et al., 2016), $\gamma_{m}^{\prime }$ was set to 1 here.

### 2.7 Integrated similarity of miRNAs

Since there may be no functional similarity between two miRNAs, we integrated the miRNA functional similarity and the GIPK similarity of miRNA m(i) and m(j) . Inspired by previous works (Dai et al., 2022), the integrated miRNA similarity between m(i) and m(j) was defined as follows:

$$\begin{array}{c} IMS\left(m\left(i\right),m\left(j\right)\right)=\left\{ \begin{array}{c} FS\left(m\left(i\right),m\left(j\right)\right),\;FS\left(m\left(i\right),m\left(j\right)\right)\neq 0\\ GM\left(m\left(i\right),m\left(j\right)\right),\;FS\left(m\left(i\right),m\left(j\right)\right)=0 \end{array}\right. \end{array}$$ (14)

### 2.8 Sample representation

Here, a miRNA-disease pair was taken as a sample. The feature vector of disease d(i) was defined as follow:

$$\begin{array}{c} FD\left(d\left(i\right)\right)=\left(IDS\left(d\left(i\right),d\left(1\right)\right),IDS\left(d\left(i\right),d\left(2\right)\right),\ldots ,IDS\left(d\left(i\right),d\left(N_{d}\right)\right)\right) \end{array}$$ (15)

Similarly, the feature vector of miRNA m(j) was defined as follow:

$$\begin{array}{c} FM\left(m\left(j\right)\right)=\left(IMS\left(m\left(j\right),m\left(1\right)\right),IMS\left(m\left(j\right),m\left(2\right)\right),\ldots ,IMS\left(m\left(j\right),m\left(N_{m}\right)\right)\right) \end{array}$$ (16)

Then, the feature vector of a sample ( d(i) , m(j) ) was defined as follow:

$$\begin{array}{c} F\left(d\left(i\right),m\left(j\right)\right)=\left(FD\left(d\left(i\right)\right),FM\left(m\left(j\right)\right)\right) \end{array}$$ (17)

**FIGURE 1:**
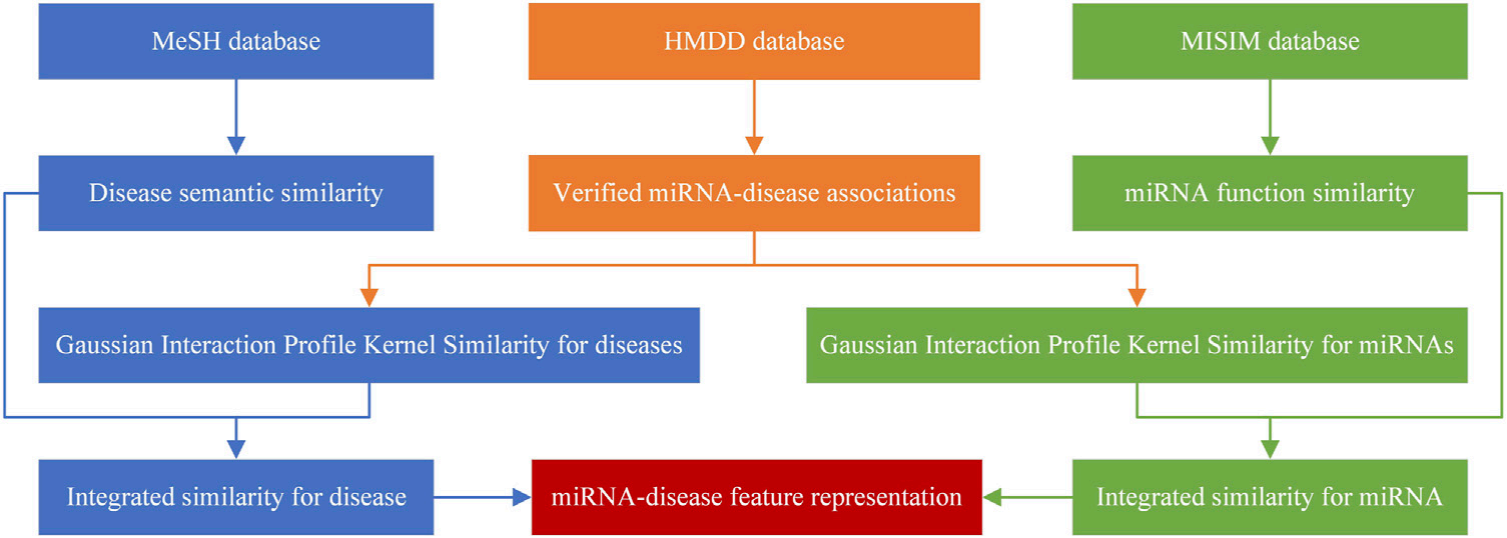
The method of sample representation.

The method of sample representation is shown in Figure 1.

### 2.9 Constructing negative sample set

In this work, the 5,430 experimentally confirmed miRNA-disease associations were taken as positive samples and the 184,155 unverified miRNA-disease pairs as unlabeled samples. Most methods (Yao et al., 2019; Zhao et al., 2019; Zhou et al., 2020; Chen et al., 2021; Li et al., 2021; Dai et al., 2022) of constructing negative sample set are to randomly select some unlabeled samples as negative samples, or apply k-means clustering on the unlabeled samples and sample negative examples from the resulted clusters. However, these methods may introduce potential positive samples into negative sample set and lead to the performance degradation of the trained model (Chen et al., 2021). Here, we proposed a novel and effective method to construct negative sample set from the total sample set. Firstly, we defined the positive sample set P , and the unlabeled sample set U :

$$\begin{array}{c} P=\left\{F\left(d\left(i\right),m\left(j\right)\right)|MD\left(m\left(j\right),d\left(i\right)\right)=1\right\} \end{array}$$ (18)

$$\begin{array}{c} U=\left\{F\left(d\left(i\right),m\left(j\right)\right)|MD\left(m\left(j\right),d\left(i\right)\right)=0\right\} \end{array}$$ (19)

And we defined the total sample set T as follows:

$$\begin{array}{c} T=P\cup U \end{array}$$ (20)

Secondly, according to the hypothesis that in the total sample set, the smaller the Minkowski distance between the two samples, the more likely they are to be the same kind of samples (Hartigan & Wong, 1979), we clustered T into K clusters by the MiniBatchKMeans (Pedregosa et al., 2011). The formula for calculating Minkowski distance was as following Eq. 21.

$$\begin{array}{c} D_{mk}\left(x,y\right)={\left(\overset{n}{\underset{u=1}{\sum }}{\left|x_{u}-y_{u}\right|}^{p}\right)}^{\frac{1}{p}} \end{array}$$ (21)

MiniBatchkmeans is an optimization of K-Means algorithm. It uses mini-batches to reduce the amount of computation required to converge to a local solution, thereby reducing the computing time required for clustering the large-scale dataset. To ensure the accuracy of clustering results, we repeated clustering ten times. Then, we denoted the K clusters as follows:

C(1),C(2),…,C(K) (22)

The proportion of positive samples in the i th cluster was defined as follows:

$$\begin{array}{c} p\left(i\right)=\frac{\left|C\left(i\right)-U\right|}{\left|C\left(i\right)\right|},\;i\in \left\{1,2,\ldots ,K\right\} \end{array}$$ (23)

Thirdly, we ranked all clusters by p(i) , and then denoted the top n (n<K) clusters with the fewest p(i) as follows:

C(h(1)),C(h(2)),…,C(h(i)),…C(h(n)) (24)

Here, C(h(i)) represents the cluster with the i th fewest p(i) .

Finally, we defined the i th negative sample set NS(h(i)) as follows:

$$\begin{array}{c} NS\left(h\left(i\right)\right)=C\left(h\left(i\right)\right)-P,i\in \left\{1,2,\ldots ,n\right\} \end{array}$$ (25)

Here, NS(h(i)) represents the cluster C(h(i)) after removing the positive sample.

Then, we constructed the total negative sample set N as follows:

$$\begin{array}{c} N=NS\left(h\left(1\right)\right)\cup NS\left(h\left(2\right)\right)\cup \ldots NS\left(h\left(n\right)\right) \end{array}$$ (26)

**FIGURE 2:**
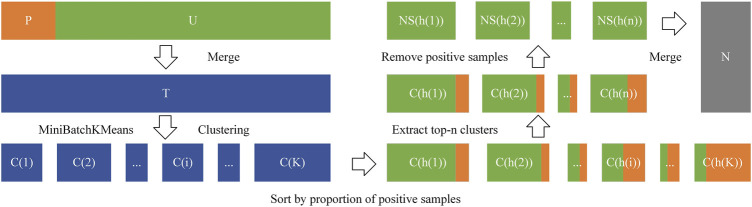
The method of constructing a negative sample set.

The number of samples in the negative sample N set constructed by the above method is 119,659. The method of constructing a negative sample set is shown in Figure 2.

### 2.10 Ensemble learning framework

**FIGURE 3:**
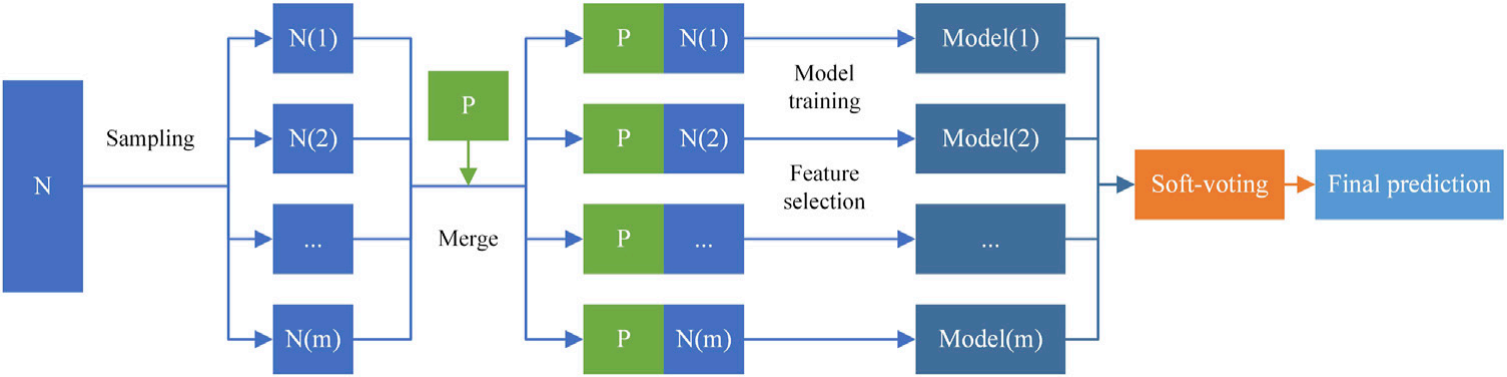
Ensemble learning framework.

In this work, we implemented an ensemble learning framework for miRNA-disease association prediction. Inspired by the previous research (Chen et al., 2019b; Dai et al., 2020; Sherazi et al., 2021; Wang et al., 2021; Zeng et al., 2021), we built the CSMDA through the following three stages: 1) construct multiple training subsets to increase the diversity of base classifiers by randomly sampling from N ; 2) perform the random forest-based feature selection to reduce noise and redundant information in the high-dimensional feature space; 3) use soft voting strategy to integrate the prediction results of all base classifiers. The process of constructing the ensemble learning framework is shown in Figure 3.

#### 2.10.1 Constructing training subsets

In this work, we constructed multiple different training subsets and balanced them to improve the prediction performance of the CSMDA. On the one hand, the diversity of subsets makes base classifiers discrepant from each other and improves the generalization ability of the CSMDA. On the other hand, multiple disparate training subsets can make full use of all negative samples. Here, we defined the size of the P as |P| . First, all samples in P were regarded as positive samples. Second, the |P| negative samples were randomly sample from N . Finally, the positive and negative samples were combined into each training subset. In this work, we constructed ten training subsets through the above methods for the CSMDA.

#### 2.10.2 Feature selection on each training subset

In the CSMDA, each miRNA-disease feature vector has 878 dimensions, which may contain a large amount of noise and redundant information. Inspired by previous research (Yao et al., 2019; Dai et al., 2022), we performed feature selection based on random forest variable importance score on each training subset. First, we trained a random forest model on each training subset and sorted all features by the variable importance scores which were generated by the random forest. Then, we selected the top X features with the highest variable importance scores to form a new feature space for each subset.

#### 2.10.3 Soft voting strategy

In this work, the Extreme Gradient Boosting (XGBoost) (Chen & Guestrin, 2016) was used as base classifier. Here, let m represent the number of training subsets. Take an unknown miRNA-disease pair as sample input, *m* base classifiers could produce *m* prediction result for the sample, and then the m prediction results were integrated by the soft voting strategy (Sherazi et al., 2021; Wang et al., 2021; Zeng et al., 2021). Specifically, the output of the i th sample by soft voting was defined as follows:

$$\begin{array}{c} O\left(i\right)=\frac{1}{m}\overset{m}{\underset{j=1}{\sum }}O\left(i,j\right) \end{array}$$ (27)

Here, O(i,j) represents the prediction scores of the j th classifier for the i th sample. If O(i)>0.5 , the miRNA-disease pair were regarded to be associated; otherwise, it was considered to be not associated.

## 3 Results

### 3.1 Performance evaluation criteria

In this work, we employed five-fold cross-validation to evaluate the performance of the CSMDA. Firstly, we adopted the known 5,430 miRNA-disease association pairs as positive samples and randomly selected an equal number of samples from the negative sample set N as negative samples. Then, all positive samples and all negative samples were combined into a sample set. Next, the constructed sample set was divided into five parts, and in each cross-validation, one part was taken out and merged with unlabeled samples to make up the test sample set, and the remaining four parts were all used as the training sample set. Here, we evaluated the CSMDA by five metrics: Precision, Recall, F1-score, AUC (Area under the receiver operating characteristic curve) and AUPR (Area under the precision-recall curve). The receiver operating characteristic (ROC) curves were obtained by plotting the true positive rate (TPR) and false-positive rate (FPR) under different levels of thresholds, and then the area under of ROC (AUC) was computed (Hajian-Tilaki, 2013). The higher the turning point of the ROC curve to the upper left, the closer the AUC is to 1, indicating the better performance of the model. The formulae for computing TPR and FPR were as following Eq. 28 and Eq. 29.

$$\begin{array}{c} TPR=\frac{TP}{TP+FN} \end{array}$$ (28)

$$\begin{array}{c} FPR=\frac{FP}{FP+TN} \end{array}$$ (29)

The Precision-Recall (PR) curves were obtained by plotting the Precision and Recall rates under different levels of thresholds, and then the area under of PR curve (AUPR) was computed (Saito & Rehmsmeier, 2015). Similarly, the higher the turning point of the PR curve to the upper right, the closer the AUPR is to 1, indicating that the model has a better performance in predicting. The formulae for computing Precision and Recall were as following Eq. 30 and Eq. 31.

$$\begin{array}{c} Precision=\frac{TP}{TP+FP} \end{array}$$ (30)

$$\begin{array}{c} Recall=\frac{TP}{TP+FN} \end{array}$$ (31)

Furthermore, F1-Score, as a comprehensive metric, is a toned-down average of precision and recall and is used to balance the effects of precision and recall and evaluate a classifier more comprehensively. In addition, the Accuracy is the result of the correct classification of the response model. The F1-Score and Accuracy can be calculated as Eq. 32 and Eq. 33 as followed.

$$\begin{array}{c} F1\mathrm{-s}core=\frac{2*Precision*Recall}{Precision+Recall} \end{array}$$ (32)

$$\begin{array}{c} Accuracy=\frac{TP+TN}{TP+TN+FP+FN} \end{array}$$ (33)

### 3.2 Performance analysis of clustering

In constructing the negative sample set, the number of clusters *K* is the key factor affecting the effectiveness of the final clustering. In this work, the silhouette coefficient (SC) (Rousseeuw, 1987) was adopted as the cluster validity index to evaluate the validity of clustering results with different cluster numbers. The silhouette coefficient is a kind of internal index to judge criteria of clustering result and it is calculated as follows:

$$\begin{array}{c} SC\left(o\right)=\frac{b\left(o\right)-a\left(o\right)}{\max\left\{a\left(o\right),b\left(o\right)\right\}} \end{array}$$ (34)

**FIGURE 4:**
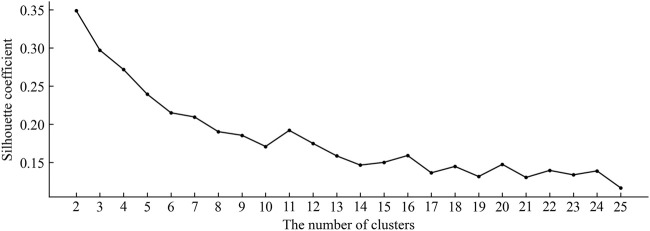
The silhouette coefficient of clustering results under different numbers of clusters.

Here, a(o) represents the average distance between sample o and other samples in its cluster, and b(o) represents the minimum average distance between sample o and samples in other clusters. The value of SC(o) ranges from -1 to 1, and SC(o) getting closer to 1 indicates that the cluster algorithm works better. First, T was divided into 2, 3 … 24, and 25 clusters by MiniBatchKMeans clustering. Then, according to each sample and its label obtained through clustering, the silhouette coefficient was calculated in turn. The silhouette coefficient with a different number of clusters is shown in Figure 4. As one can see, the silhouette coefficient decreases gradually with the increase of the number of clusters and achieves a maximum of 0.349 when the number of clusters is 2. Therefore, we set the values of *K* to 2 in the CSMDA.

### 3.3 Performance analysis of base classifier

Base classifier plays an importance role in the prediction performance of the ensemble learning framework. In this work, we compared the performance of four base classifiers: AdaBoost, Random Forest (RF), Extreme Gradient Boosting (XGBoost) and Extremely Randomized Trees (ExtRa Trees). For optimal performance, we optimized the hyper-parameters of each model. The prediction performance of the CSMDA using different base classifiers are listed in Table 1. As one can see, the Precision of the XGBoost is 0.9674, the Recall is 0.9543, the F1-score is 0.9608, the AUROC is 0.9927 and the AUPR is 0.9939. The XGBoost is lower than the RF in terms of Precision, but it is higher than other models in all other metrics. Therefore, the XGBoost was employed in the CSMDA.

**TABLE 1:** Performance comparison of the CSMDA using different base classifiers.

Model	Precision	Recall	F1-score	AUROC	AUPR
CSMDA-AB	0.9567	0.9267	0.9414	0.9885	0.9901
CSMDA-ERT	0.9666	0.9514	0.9589	0.9907	0.9926
CSMDA-RF	**0.97**	0.9468	0.9582	0.9912	0.9929
CSMDA-XGB	0.9674	**0.9543**	**0.9608**	**0.9927**	**0.9939**

### 3.4 Feature dimension analysis of samples

**FIGURE 5:**
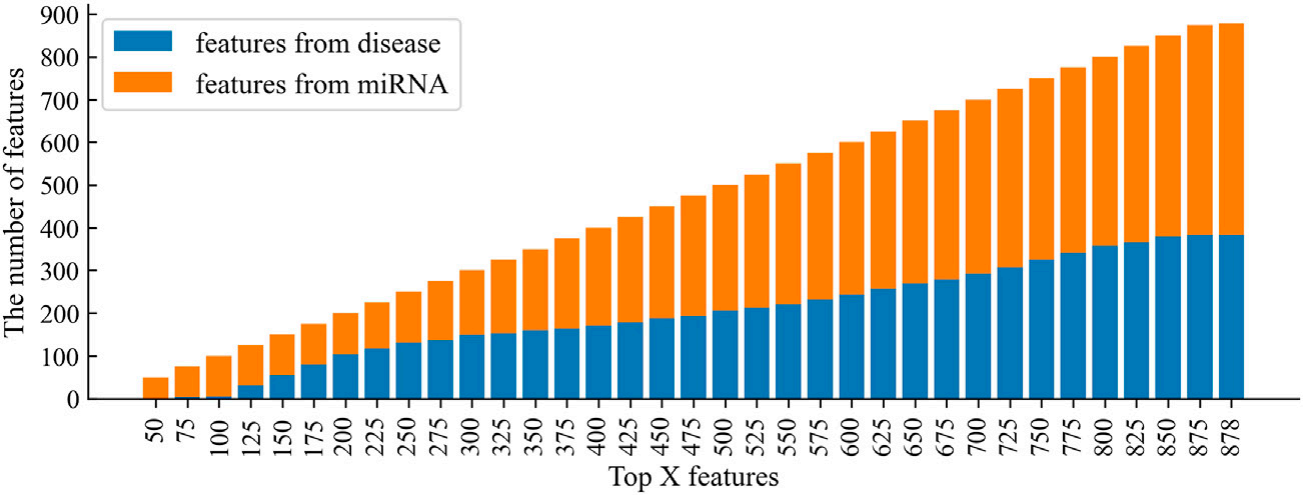
The distribution of features from miRNAs and diseases among the top X features.

In the feature selection, according to the variable importance scores, 100, 75, 50, and 25% features were selected from the original feature space to construct the training set, denoted as CSMDA-NOFS, CSMDA-FS75, CSMDA-FS50, and CSMDA-FS25, respectively. Then, we evaluated the prediction performance of the CSMDA with different number of features, and the results were listed in Table 2. As one can see, when the dimension of the training sample is 75% of the length of the original feature vector, the effect of feature selection on improving the performance of the CAMDA is optimum. Therefore, we set the feature dimension of the training set to 75% of the length of the original feature vector. We further analyzed the contribution of miRNA and disease to the feature vector, the distribution of features from miRNAs and diseases among the X features with the highest variable importance scores is shown in Figure 5. As we can see from Figure 5, the number of features from miRNAs is generally greater than that from diseases, which is consistent with the fact that the number of miRNAs is greater than that from the diseases. This indicates that feature selection based on the variable importance score is reasonable.

**TABLE 2:** Performance comparison of the CSMDA under different dimension training samples.

Model	Precision	Recall	F1-score	AUROC	AUPR
CSMDA-NOFS	0.9674	0.9543	0.9608	0.9927	0.9939
CSMDA-FS75	**0.9676**	0.9545	**0.9610**	**0.9928**	**0.9940**
CSMDA-FS50	0.9667	**0.9551**	0.9608	0.9927	0.9939
CSMDA-FS25	0.9657	0.9540	0.9598	0.9916	0.9930

### 3.5 Performance comparison between clustering-based sampling method for miRNA-disease association prediction and other miRNA-disease association prediction models

To prove the ability of the CSMDA to predict potential disease-associated miRNAs, we compared it with six state-of-the-art MDA prediction models, including ABMDA (Zhao et al., 2019), ANMDA (Chen et al., 2021), GAEMDA (Li et al., 2021), GBDT-LR (Zhou et al., 2020), IRFMDA (Yao et al., 2019) and ERMDA (Dai et al., 2022). First, the CSMDA and other MDA prediction models constructed negative sample set by their respective methods. Secondly, we used the recommended hyper-parameters for these models. Finally, we performed 500 times five-fold cross-validation for each model. The performance of the above MDA prediction models are shown in Table 3. As one can see, the Precision, Recall, F1-score, AUC and AUPR of the CSMDA is 0.9676 ± 0.0052, 0.9545 ± 0.0059, 0.9610 ± 0.0042, 0.9928 ± 0.0012, and 0.9940 ± 0.0009 respectively, which superior to other methods in all metrics. The results proved the outstanding prediction performance of the CSMDA.

**TABLE 3:** Performance comparison of the CSMDA with other MDA prediction models.

Model	Precision	Recall	F1-score	AUROC	AUPR
ABMDA [19]	0.8213 ± 0.0033	0.8371 ± 0.0044	0.8290 ± 0.0030	0.9023 ± 0.0021	0.8879 ± 0.0032
ANMDA [22]	0.8561 ± 0.0017	0.8728 ± 0.0020	0.8643 ± 0.0014	0.9373 ± 0.0005	0.9328 ± 0.0008
GAEMDA [21]	0.8146 ± 0.0031	0.9111 ± 0.0028	0.8597 ± 0.0010	0.9352 ± 0.0001	0.8850 ± 0.0010
GBDT-LR [20]	0.8403 ± 0.0026	0.8567 ± 0.0031	0.8484 ± 0.0021	0.9246 ± 0.0010	0.9177 ± 0.0015
IRFMDA [18]	0.8447 ± 0.0021	0.8598 ± 0.0025	0.8521 ± 0.0016	0.9267 ± 0.0009	0.9222 ± 0.0012
ERMDA [23]	0.8740 ± 0.0039	0.9043 ± 0.0019	0.8889 ± 0.0022	0.9561 ± 0.0013	0.9542 ± 0.0020
CSMDA	**0.9676 ± 0.0052**	**0.9545 ± 0.0059**	**0.9610 ± 0.0042**	**0.9928 ± 0.0012**	**0.9940 ± 0.0009**

### 3.6 Case studies

To prove the application value of the CSMDA in guiding biological experiments, we performed case studies on three common cancers, including breast cancer, colon cancer and lung cancer. Firstly, we combined the 5,430 positive samples verified by the experiment and the 5,430 negative samples randomly selected from the negative sample set N into the training set of CSMDA. Secondly, we identified the positive and negative samples to which the three diseases belong. Thirdly, in the case study of current cancer, remove all samples related to current cancer in the training set. Finally, we trained CSMDA on this training set, and scored miRNA-disease pairs related to current cancer by using the CSMDA. We verified the top 20 miRNAs predicted to be associated with each cancer, and the results were listed in Table 4. Here, we validated these predicted miRNAs through the dbDEMC (Database of differentially expressed miRNAs in human cancers) database (Yang et al., 2017) or literatures. As one can see from Table 4, for breast cancer and lung cancer, all predicted miRNAs were confirmed by the dbDEMC database; for colon cancer, all predicted miRNAs except hsa-mir-34c were confirmed by the dbDEMC database. However, Hiyoshi et al. demonstrated that the expression level of Mir-34C in human colon cancer cells was higher than that in non-tumor cells (Hiyoshi et al., 2015). In summary, case study demonstrated that the CSMDA was reliable for predicting disease-associated miRNAs.

**TABLE 4:** The top 20 miRNAs for three cancers predicted by the CSMDA.

Disease	Rank	miRNA	Evidence
breast cancer	1	hsa-mir-195	dbDEMC
	2	hsa-mir-146a	dbDEMC
	3	hsa-mir-24	dbDEMC
	4	hsa-let-7e	dbDEMC
	5	hsa-mir-9	dbDEMC
	6	hsa-mir-219	dbDEMC
	7	hsa-mir-148a	dbDEMC
	8	hsa-mir-218	dbDEMC
	9	hsa-let-7a	dbDEMC
	10	hsa-mir-29a	dbDEMC
	11	hsa-mir-223	dbDEMC
	12	hsa-mir-30d	dbDEMC
	13	hsa-mir-92a	dbDEMC
	14	hsa-mir-210	dbDEMC
	15	hsa-mir-200c	dbDEMC
	16	hsa-mir-17	dbDEMC
	17	hsa-mir-214	dbDEMC
	18	hsa-mir-372	dbDEMC
	19	hsa-mir-106b	dbDEMC
	20	hsa-mir-221	dbDEMC
colon cancer	1	hsa-mir-24	dbDEMC
	2	hsa-mir-20a	dbDEMC
	3	hsa-mir-125b	dbDEMC
	4	hsa-mir-182	dbDEMC
	5	hsa-mir-29a	dbDEMC
	6	hsa-mir-214	dbDEMC
	7	hsa-mir-17	dbDEMC
	8	hsa-mir-21	dbDEMC
	9	hsa-mir-30b	dbDEMC
	10	hsa-mir-29b	dbDEMC
	11	hsa-mir-19b	dbDEMC
	12	hsa-mir-19a	dbDEMC
	13	hsa-mir-18a	dbDEMC
	14	hsa-mir-141	dbDEMC
	15	hsa-mir-155	dbDEMC
	16	hsa-mir-223	dbDEMC
	17	hsa-mir-127	dbDEMC
	18	hsa-mir-34c	Hiyoshi, Y., et al. [40]
	19	hsa-mir-1	dbDEMC
	20	hsa-mir-126	dbDEMC
lung cancer	1	hsa-mir-29c	dbDEMC
	2	hsa-mir-92a	dbDEMC
	3	hsa-mir-206	dbDEMC
	4	hsa-mir-214	dbDEMC
	5	hsa-mir-183	dbDEMC
	6	hsa-mir-210	dbDEMC
	7	hsa-mir-142	dbDEMC
	8	hsa-mir-221	dbDEMC
	9	hsa-mir-30e	dbDEMC
	10	hsa-mir-24	dbDEMC
	11	hsa-mir-223	dbDEMC
	12	hsa-mir-20b	dbDEMC
	13	hsa-mir-193b	dbDEMC
	14	hsa-mir-191	dbDEMC
	15	hsa-mir-22	dbDEMC
	16	hsa-mir-124	dbDEMC
	17	hsa-mir-18b	dbDEMC
	18	hsa-mir-30a	dbDEMC
	19	hsa-mir-148a	dbDEMC
	20	hsa-mir-15b	dbDEMC

## 4 Conclusion

In this work, we presented a clustering-based sampling method for predicting miRNA-disease associations, named CSMDA. Firstly, the CSMDA integrated similarity of disease and miRNA to represent samples. Secondly, the CSMDA implemented an effective clustering-based sampling method to construct negative sample set. Thirdly, the CSMDA employed a random forest-based feature selection method to reduce noise and redundant information in the high-dimensional feature space. Finally, the CSMDA implemented an ensemble learning framework for predicting miRNA-disease associations by soft voting. The experimental results and case studies on the three cancers demonstrate that the CSMDA is a reliable model to predict disease-associated miRNAs. The main contribution of the CSMDA is to propose a new method to construct a more effective negative sample set, which avoids the possibility of introducing potential positive samples into negative sample set as much as possible. The negative sample set constructed by our method not only makes CSMDA perform well, but also improves the performance of other MDA prediction models. However, it should be noted that there are several limitations to the CSMDA. First, it is still inevitable to introduce potential positive samples in the stage of constructing the negative sample set. Second, the clustering algorithm used in the CSMDA is MiniBatchKMeans which showed good clustering effect, but other clustering algorithms may make the negative sample set purer. We will study the clustering effect of other clustering algorithms on the total sample set in the next work. Finally, in current work, the information associated with miRNA and disease is limited, which may result in the essential features that are helpful to identify miRNA-disease associations not being extracted in the CSDMA. In the future, we will integrate more features related to disease and miRNA into the CSMDA. In summary, we hope that the CSMDA can help researchers make breakthroughs in the treatment of complex human diseases at the miRNA level.

## Data Availability

The original contributions presented in the study are included in the article/Supplementary Material, further inquiries can be directed to the corresponding author.
